# Acute Rheumatic Fever and Rheumatic Heart Disease: Highlighting the Role of Group A Streptococcus in the Global Burden of Cardiovascular Disease

**DOI:** 10.3390/pathogens11050496

**Published:** 2022-04-21

**Authors:** Tangeni Auala, Ben’Lauro Goncalves Zavale, Amam Çhinyere Mbakwem, Ana Olga Mocumbi

**Affiliations:** 1 Division of Adult Cardiology, Windhoek Central Hospital, Windhoek 9000, Namibia; tangeni.auala@alumni.uct.ac.za; 2 Faculty of Medicine, Universidade Eduardo Mondlane, Maputo 1113, Mozambique; zavale1978@gmail.com; 3 Department of Medicine, College of Medicine, University of Lagos, Lagos 101017, Nigeria; ambakwem@hotmail.com; 4 Instituto Nacional de Saúde, Marracuene 1220, Mozambique

**Keywords:** Group A Streptococcus, rheumatic fever, rheumatic heart disease, cardiovascular morbidity and mortality

## Abstract

Group A Streptococcus (GAS) causes superficial and invasive infections and immune mediated post-infectious sequalae (including acute rheumatic fever/rheumatic heart disease). Acute rheumatic fever (ARF) and rheumatic heart disease (RHD) are important determinants of global cardiovascular morbidity and mortality. ARF is a multiorgan inflammatory disease that is triggered by GAS infection that activates the innate immune system. In susceptible hosts the response against GAS elicits autoimmune reactions targeting the heart, joints, brain, skin, and subcutaneous tissue. Repeated episodes of ARF—undetected, subclinical, or diagnosed—may progressively lead to RHD, unless prevented by periodic administration of penicillin. The recently modified Duckett Jones criteria with stratification by population risk remains relevant for the diagnosis of ARF and includes subclinical carditis detected by echocardiography as a major criterion. Chronic RHD is defined by valve regurgitation and/or stenosis that presents with complications such as arrhythmias, systemic embolism, infective endocarditis, pulmonary hypertension, heart failure, and death. RHD predominantly affects children, adolescents, and young adults in LMICs. National programs with compulsory notification of ARF/RHD are needed to highlight the role of GAS in the global burden of cardiovascular disease and to allow prioritisation of these diseases aimed at reducing health inequalities and to achieve universal health coverage.

## 1. Introduction

*Streptococcus pyogenes*, also referred to as Group A Streptococcus (GAS), has been recognized as an important cause of global morbidity and mortality, especially in resource poor settings and is said to be among the top 10 causes of death worldwide [1,2]. GAS causes a wide range of diseases including superficial infection (scarlet fever, impetigo, pharyngitis), invasive infection (cellulitis, necrotizing fasciitis, skeletal infections, sepsis, toxic shock syndrome), and immune mediated post infectious sequalae (acute rheumatic fever/rheumatic heart disease, post-streptococcal glomerulonephritis, and recently paediatric autoimmune neuropsychiatric disorders associated with streptococcal infections (PANDAS) [3,4,5]. 

The traditionally accepted route of transmission of *Streptococcus pyogenes* is by heavy respiratory droplets. However, there is recent evidence that other routes may play an important role in the transmission: oronasal secretions, small airborne particles, skin-to-skin contact, surfaces, bedding and fabrics, food, and insects [6,7]. Though spread is from infectious individuals, some studies suggest that transmission from carriers may be of epidemiological importance [8,9,10].

Although acute rheumatic fever (ARF) and its sequela rheumatic heart disease (RHD) virtually disappeared in high income settings by late 20th century, they remain major public health concerns in low- and middle-income countries (LMICs) and in vulnerable communities in high income countries [11]. Renewed interest in these diseases resulted in more research, action, and advocacy by groups from affected regions which revealed undeniable evidence that ARF/RHD is the most common acquired heart disease among children and young adults with a female preponderance [12]; it still carries a high risk of death and disability from heart failure, stroke, and infective endocarditis, and constitutes an enormous socioeconomic stress in endemic regions(LMICs) [13,14,15]. In 2013 the World Heart Federation released a position statement on the prevention and control of RHD with a goal to reduce premature deaths caused by RHD in individuals younger than 25 years of age by 25% by the year 2025 [16]. A milestone was achieved in 2018 when ARF and RHD were declared a global health priority for the first time with the passage of a Global WHO Resolution on Rheumatic Fever and Rheumatic Heart Disease. This important resolution establishes key actions, mobilises resources, and renews the global commitment toward the eradication of ARF [17].

## 2. Epidemiology

Accurate estimates of ARF and RHD disease burden are lacking due to a paucity of comprehensive disease registries, reliance on passive surveillance systems, and the underreporting of both acute and chronic cases from endemic areas for *Streptococcus pyogenes* infections [18]. Serious GAS disease was previously estimated to affect about 18.1 million people each year, with around 1.78 million new cases and 517,000 deaths annually [19,20]. More recent estimates put the global burden of RHD at 33.4 million and annual mortality around 639,000 [11].

Prevalence of non-invasive GAS infection varies according to the geographical location and seasons; pharyngitis is dominant in temperate regions during the winter months, while impetigo is more common in the tropical zones during summer [21]. Children are both the reservoir of GAS (represent the pool for infection of adults) and the target population for pharyngitis, invasive, and non-invasive infections. The peak age incidence for infections is from 5 to 15 years, with infections in older ages usually occurring in the setting of large gatherings of people in close quarters [21,22].

Developed countries have reported a decreased incidence in invasive GAS disease. Wahl et al. reported a low incidence rate of invasive *Streptococcus pyogenes* disease between 1996 and 2002 from a voluntary laboratory surveillance system [23]. Germany is classified as a non-endemic country as less than 0.15 deaths per 100,000 population among children 5 to 9 years of age were due to RHD [11]. Despite the reduced incidence of GAS in high income countries, recent resurgences have been reported likely related to changes in circulating strains and/or host susceptibility [24,25]. In 2015, the United States of America reported over 15,000 cases of invasive GAS infection while Canada reported an increase of 5.24 cases over the 2.9 cases/100,000 incidence in 2003 [24,26,27]. A more recent systematic review and meta-analysis on the incidence of sore throat and GAS pharyngitis in children at high risk of developing ARF worldwide found that the pooled incidence rates for sore throat in children at risk of developing ARF in these regions at 82.5 per 100 child-years [28]. This rate is higher than rates reported in developed countries (32.70–40 per 100 child-years); for GAS pharyngitis the incidence rates were 10.8 per 100 child-years (similar to 12.8–14 per 100 years in developed countries) [28]. In East Africa, Bimerwe et al. were able to include 13 articles in their systematic review and meta-analysis, finding a pooled prevalence of RHD also considerably higher than the results from developed countries, at 17.9 per 1000 children (95% CI = 11.6–24.2) [29]. Thus, overall GAS infections are worse in socioeconomically disadvantaged groups in developing and developed nations [25,26,27].

## 3. Natural History

The natural history and progression of ARF and RHD remain incompletely understood. Evidence supports the view that ARF results from an autoimmune response to pharyngeal infection with GAS in genetically predisposed individuals, which is mediated through molecular mimicry [30,31]. About 0.3–3% of people with GAS pharyngitis develop ARF, depending on genetic predisposition and the virulence of the infecting strain [19,32,33]. Whether the finding of more severe forms of RHD at younger ages in Sub Saharan Africa is related to genetic background or repeated GAS infections in the context of health systems challenges is still to be revealed as is the possible role of GAS skin infection causing ARF in LMICs, another important consideration [13,33]. 

## 4. Pathogenesis

The initiation of the host-GAS interaction is by avid adhesion of the bacteria via multiple adhesins [1,31,33]. In addition to adhesion and colonisation, intracellular invasion can occur and this requires the expression of M protein and/or fibronectin-binding proteins such as SfbI by GAS [34,35,36]. Internalisation may lead to carriage and persistence as the GAS M protein and hyaluronic acid capsule enables the organism to evade host defence mechanisms (viz opsonization and phagocytosis) [1,35]. 

### 4.1. Molecular Mimicry

The transition from a bacterial infection to tissue damage resulting in ARF/RHD is through the immune response to the superficial infections. The antibody and cellular immune response directed against GAS antigens cross reacts with tissues in the heart, joints, brain, skin, and subcutaneous tissues of the susceptible host. This molecular mimicry is due to structural similarity (shared epitopes) between the host tissues and GAS antigens. Studies have demonstrated cross reactive antigens on the GAS cell wall, cell membrane, and hyaluronate capsule that react with three major human host subsets, mainly N-acetyl-glucosamine, myosin, and related molecules and DNA [30,37,38]. 

The proposed events in rheumatic carditis and valvulitis are both cellular and antibody mediated. The major target of cross-reactive GAS polysaccharide directed antibodies is the valve endothelium and lamina, though there is also reaction with myocardial myosin [34]. This antibody cross reactivity leads to inflammation at the valve surface and expression of increased amounts of the adhesion molecule VCAM-1 (Vascular cell adhesion molecule 1). This promotes the binding, infiltration, and extravasation of cross-reactive T-cells; these T-cells have cross-reactivity with streptococcal M proteins and similar host alpha helical protein antigens (e.g., myosin, laminin, tropomyosin, or vimentin). The T-cells differentiate into CD4+ TH1 cells producing gamma interferon that causes scarring and fibrosis and IL-17A that promotes neovascularization in the normally avascular valve tissue [38,39]. These processes predispose the valve to cellular infiltration through both the activated valve endocardial surface and the neovascularized scar tissue. Antibodies to collagen have also been demonstrated in RHD and may contribute to valve damage, but this pathway will be activated only once the valve is already damaged and underlying collagen is exposed [30]. Aschoff bodies are the typical histopathological lesions in RHD.

#### Pharyngitis versus Impetigo in Immune Mediated Sequalae

Genetic and epidemiological evidence for skin infection as the event that leads to ARF is mounting, but pharyngeal infection is still considered to be the trigger in most cases [13,40,41,42]. Earlier studies have shown consistent elevation of Anti Streptolysin O (ASO) titres in GAS pharyngitis, while in patients with GAS impetigo inconsistent elevations were seen [40]. Based on the antigenicity of the 3’ terminal repeat region of *emm*, phage typing with M proteins is classified into I and II; with only class I associated with ARF. The *emm* chromosomal arrangement was then further classified into 5 patterns responsible for different manifestations: pattern A–C (class I) causing pharyngitis; pattern D (class I) causing impetigo; and pattern E (class II) causing both pharyngitis and impetigo [40,41]. These data, derived from the United States of America, are under debate because in populations from areas with the highest burden of RHD (e.g., native Australians, New Zealanders, and Fijians), GAS impetigo is more frequent than pharyngitis [42,43,44]. The reasons for this could include: the diversity of GAS species between the tropical and temperate regions; possible coinfection by strains causing both impetigo and pharyngitis; and priming by impetigo strain for the immune reaction with a pharyngitis [40]. The possibility of GAS skin infection as the trigger for ARF has great implications for the control programmes for ARF/RHD which have previously only focused on prevention of GAS pharyngitis.

### 4.2. Genetic Susceptibility

The heritable genetic susceptibility to ARF is demonstrated by the increased risk of concordance among monozygotic twins over dizygotic twins (44% vs. 12%) [19]. The lifetime cumulative incidence of ARF in populations exposed to rheumatogenic GAS infection is consistently 3–6% irrespective of ethnicity or geography [45]. The familial aggregation of rheumatic fever as originally reported by Cheadle, states that the chance of an individual with a family history of ARF acquiring the disease is 5 times greater than that of an individual who has no family history. This has been supported by a study of children raised separately from parents with RHD, who had a relative risk of 2.93 for the development of rheumatic fever compared with children whose parents did not have RHD [46]. Twin studies have evaluated the extent to which the familial occurrence of ARF is due to genetic and environmental factors. Phenotypic concordance among dizygotic twins suggests that ARF has a non-Mendelian inherited component. The risk of rheumatic fever in a monozygotic twin when the co-twin previously had rheumatic fever is more than six times greater than that in dizygotic twins. The heritability of rheumatic fever is 60%, highlighting heredity as a major susceptibility factor of the disease [47].

Several genes responsible for the innate and adaptive immune response, cytokines, and B-cell alloantigens have been associated in the development of ARF and RHD [48,49,50]. A recent multicenter case-control genome-wide association study (GWAS), the Genetics of Rheumatic Heart Disease, studied more than 7 million genotyped and inputted single-nucleotide variations from 4809 African individuals [51]. This study demonstrated a new candidate susceptibility locus (11q24.1) which reached genome-wide significance in and exclusive to Black African individuals and thus a heritable component to RHD susceptibility in African individuals [51]. Although significant associations have been found between genetic factors and ARF, study outcomes either contradict each other or are not reproducible. The discovery of the specific genetic and heritable factors for ARF would allow for screening of such variants and identification of individuals who are at high risk and would derive benefit from primary penicillin prophylaxis or vaccination against GAS [52].

### 4.3. Other Factors

Socioeconomic status (household income, level of education, unemployment) affects multiple potential risk factors for the development of ARF and RHD [53,54,55]. Potential risk/protective factors that have been identified and demonstrated include environmental factors (number of social contacts, household crowding and bed sharing, household resources, laundry, housing conditions); healthcare factors (health literacy, distance to and healthcare access); and health and nutrition factors (health status, oral health status and services, nutrition) [53].

**Figure 1:**
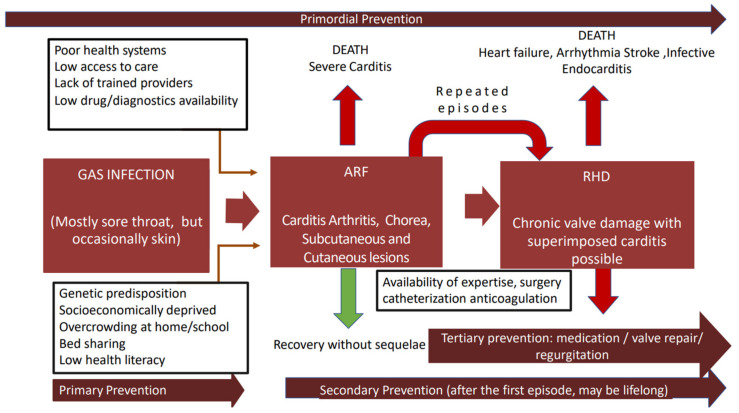
Natural history of GAS-immune mediated disease in susceptible host. The determinants of natural history of acute rheumatic fever and rheumatic heart valve disease (boxes with black outline) in susceptible individuals from highly endemic areas are presented. We highlight the possible interventions for prevention (deep red arrows). Some individuals recover from ARF with no sequelae (green arrow) As shown, fatal outcomes may result from ARF and RHD and are highlighted through red arrows; repeated episodes of ARF lead to chronic valve damage. GAS Group A Streptococcus; ARF acute rheumatic fever; RHD rheumatic heart disease.

The pathogenesis and natural history of GAS immune mediated sequalae are summarized in the Figure 1.

## 5. Clinical Features

GAS pharyngitis can be difficult to differentiate from viral infections, necessitating a high index of suspicion and laboratory testing for confirmation. ARF is a multiorgan inflammatory disorder affecting the heart (carditis), joints (arthritis and arthralgia), brain (Sydenham’s chorea), skin (erythema marginatum), and subcutaneous tissue (subcutaneous nodules). RHD is characterized by typical heart valve lesions, classified as regurgitation and stenosis that may be complicated with heart failure, arrhythmias, infective endocarditis, or thromboembolic phenomena.

Diagnosis of GAS pharyngitis is dependent on throat swabs for culture, but the presence of the organism could represent colonisation rather than infection. Isolation of organisms from skin lesions may be more difficult because of super colonisation by *Staphylococcus aureus*. Identification of serotypes using T-typing and *emm* gene analysis is still a research tool. 

Serological diagnosis of immune mediated manifestations occurring weeks after the GAS infection is based on strong host immune responses against bacterial enzymes: anti-streptolysin O (ASO), anti-DNase B, anti-hyaluronidase, anti-NADase, and anti-streptokinase [1]. ASO titre is the most used test to confirm antecedent streptococcal infection, but if ASO titres are very low, anti-DNase B or anti-hyaluronidase assays can be used to establish antecedent infection.

Diagnosis of ARF: No single clinical feature or laboratory test is diagnostic. The diagnosis of ARF is made using a combination of clinical and laboratory criteria with evidence of antecedent GAS infection after other causes of clinical presentation have been excluded [30]. 

Originally described in 1944, the Jones criteria provided a framework for defining major and minor manifestations to make a syndromic diagnosis of ARF [56]. The major criteria and main clinical presentations of ARF include arthritis (usually asymmetric migratory/fleeting polyarthritis involving the large joints predominantly) in 60–80% of patients; pancarditis (valvulitis, myocarditis and pericarditis) which can be clinical or subclinical in 50–80%; central nervous system involvement (e.g., Sydenham chorea) in 10–30%; subcutaneous nodules in 0–10%; and erythema marginatum in less than 6% [13]. The minor manifestations include arthralgia, fever, elevated acute phase reactants (ESR, CRP), and prolonged PR interval on electrocardiogram. Elevated acute phase reactants support the diagnosis: erythrocyte sedimentation rate (ESR) ≥ 60 mm/h and C-reactive protein (CRP) ≥ 3 mg/dL (≥30 mg/L); lower ranges of CRP and ESR can be seen in high-risk groups and in the setting of isolated chorea (late feature) or following anti-inflammatory treatment when acute phase reactant levels may have returned to normal. Electrocardiography (ECG) may demonstrate abnormal atrioventricular (AV) conduction with prolonged PR interval (>200ms) representing first-degree AV block being the most common abnormality. This rarely progresses to second-degree and complete heart block. 

The most recent revision of the Jones criteria in 2015 incorporated three important modifications [46,57,58]:

1. Risk stratification based on disease endemicity: The 2015 modification identifies low risk populations as those with ARF incidence <2 per 100,000 school-aged children per year or a prevalence of RHD of ≤1 per 1000 patients at any age per year. Additionally, it emphasizes that children from non low-risk ARF populations should be considered at moderate-to-high risk (moderate and high being treated equally).
2. Different categorization and implications of carditis, joint manifestations, parameters of fever and inflammation dependent on population risk stratifications.
3. The recommendation that all patients with suspected or confirmed ARF undergo doppler echocardiography and recognition of echocardiographic evidence of carditis (subclinical carditis) as a major manifestation of ARF in low-and high-risk populations, based on meta-analysis that included 23 studies from five continents demonstrating that patients with ARF have weighted pooled prevalence of subclinical carditis of 16.8%, and nearly half (44.7%) had deterioration in valve function over time [46,57,58].

These changes improve the diagnosis of ARF among moderate/high-risk populations and re-establish the Jones criteria as the international gold standard for ARF diagnosis. This revision also provides guidance on diagnosing recurrent ARF. 

Diagnosis of RHD: The clinical features of RHD are dependent on which valves (typically the left-sided valves) are affected, number of valves affected, severity of valve lesions, and presence of associated complications. RHD is a result of thickening and fibrosis of valvular apparatus and commissural fusion [59,60,61]. 

The main clinical symptoms are shortness of breath during activity, rest, or lying down; body swelling; and palpitations and chest pain. Examination features include cardiac murmurs and changes in heart sounds, features of fluid overload, and cardiac chamber enlargement. Compensatory haemodynamic changes can allow for a prolonged asymptomatic latent period [62]. The diagnosis of RHD can be made after a confirmed episode of ARF, but a significant proportion of patients—particularly those in LMIC—present without any prior symptoms or memory of prior ARF attack and their initial presentation is complicated by decompensated heart failure, arrhythmias, systemic embolism, infective endocarditis, and pulmonary hypertension; some women are diagnosed in pregnancy [12,62,63,64].

Each valve lesion in RHD is associated with clinical features that guide the assessment and disease management. Comprehensive clinical assessment includes an ECG and chest X-ray. Echocardiography is vital to confirming clinical findings, the number and severity of affected valves, and assessing the physiological consequences and complications of RHD and planning management.

Mitral regurgitation (MR). Contemporary data demonstrate that pure mitral regurgitation is the predominant lesion in childhood with mixed mitral and mixed aortic valve disease emerging as the dominant lesions in early adulthood [12,62]. Acute mitral valvulitis causes annular dilatation and chordal elongation of anterior mitral valve leaflet, fibrosis, scarring and contracture of components of the mitral valve apparatus (valve leaflets, chordae tendineae, and papillary muscles). This causes excessive tethering of posterior leaflet relative to the anterior mitral valve leaflet (pseudoprolapse of AMVL) and mitral valve regurgitation. Rarely rheumatic MR is caused by chordal rupture [63]. Symptoms vary with severity and rate of MR progression, increased left atrial, pulmonary venous and pulmonary arterial pressures, and elevated left ventricular (LV) end diastolic volumes and pressure. Most patients tolerate chronic MR so well that their first clinical presentation is that of advanced heart failure with reduced cardiac output, pulmonary congestion, or irreversible left ventricular dysfunction [61]. 

Pure mitral stenosis (MS) is rare in childhood and more common during adulthood [12]. It results from leaflet and chordal thickening, fibrosis, and fusion. As the mitral valve opening becomes restricted, there is doming of the leaflets in diastole [63,64]. With progressive narrowing and reduction of the orifice area, the left atrial pressure rises to maintain cardiac output. As in MR pulmonary hypertension, right ventricular dilation and secondary tricuspid regurgitation develop [65,66]. Symptoms of MS may arise insidiously over many years related to the aforementioned changes or may develop abruptly because of rapid heart rate (e.g., atrial fibrillation) or acute volume overload [67]. Mixed mitral valve disease with either predominant MR/MS can develop with progression of RHD. 

Aortic valve disease in RHD, like mitral valve disease, results from thickening and bowing of the leaflet tips and commissural fusion. Aortic regurgitation (AR) has a gradual onset and a prolonged asymptomatic period [14]. Isolated aortic stenosis (AS) is an uncommon manifestation of RHD and is related to a reduced aortic orifice area [14]. Survival is good during the asymptomatic phase of AS but with the development of symptoms, mortality exceeds 90% within a few years, with symptoms of heart failure and syncope associated with the worst prognosis [68].

Tricuspid valve Disease occurs as a primary valvulitis or secondary to significant left-sided heart disease. Tricuspid regurgitation is more commonly seen than tricuspid stenosis. Clinical features may not be apparent because they are obscured by concomitant left sided disease or can be rapidly modified by diuretic therapy [69]. Primary tricuspid disease has a grave impact on prognosis as it causes right heart failure and increased mortality.

Multivalve rheumatic heart disease is common and carries a high risk for ventricular dysfunction, symptomatic heart failure, and death [68]. Careful clinical evaluation supplemented by comprehensive echocardiography can discern the severity of each valve lesion and guide management [67].

### Echocardiography in Rheumatic Heart Disease

Echocardiography plays an essential role in the diagnosis and management of RHD, confirming the aetiology and determining the severity as well as the presence of haemodynamic consequences and complications of the valve lesions [66]. The minimal echocardiographic criteria to establish a diagnosis of RHD in the setting of mitral/aortic valve disease have been defined in the 2012 World Heart Federation (WHF) guidelines [70].

Echocardiography can monitor disease progression, detect latent/subclinical disease—when secondary prophylaxis with penicillin is more likely to be effective—and allow for timely catheter-based or surgical interventions [61]. Increasing access to this diagnostic tool in LMICs through task-shifting, point of care handheld devices, and abbreviated protocols can allow for earlier diagnosis of ARF and RHD and improve outcomes for patients with ARF and RHD [69]. 

## 6. RHD and Pregnancy

RHD has a female preponderance affecting women in their childbearing years and contributing up to 34% of maternal mortality in resource limited settings [12,14,71]. RHD is commonly diagnosed for the first time in pregnancy, when physiological changes can unmask valve lesions. In Uganda, echocardiographic screening of pregnant women revealed that 1.7% had cardiac disease, with less than 5% being aware of their diagnosis [72]. Delayed diagnosis of heart disease is a risk factor for maternal death, and in LMICs many women with RHD present after 20 weeks gestation or are unaware of their RHD diagnosis until haemodynamic decompensation occurs. 

Frequent complications during pregnancy include heart failure, arrythmias and thromboembolic events, which all increase the risk of poor perinatal outcomes [73]. Adverse maternal events are predicted in women with left heart obstruction, pulmonary hypertension, previous or current heart failure, need for up titration of medication, higher NYHA class (III and IV), presence of mechanical valves, maternal age older than 28 years of age or body mass index higher than 28 kg/m^2^ [74,75,76,77,78]. The Modified World Health Organization Classification of maternal cardiovascular risk (mWHO) is a risk index that includes general and lesion specific diagnoses and is considered the most accurate risk assessment tool, assigning women to one of four risk classes [75,76]. Class I represents the lowest risk category with no detectable/increased risk of maternal mortality and mild increase in maternal morbidity, while mWHO Class IV represents such increased risk of maternal morbidity and mortality that pregnancy is contraindicated, and termination of pregnancy should be discussed [75]. 

Preconception counselling with maternal cardiovascular risk stratification and provision of safe reliable contraception should be available to all women with RHD [77,78,79]. The Global RHD Registry (REMEDY) revealed that only 5% of women with prosthetic valves and 2% with severe mitral stenosis were using contraception [14]. As highlighted in the Pan-African Society of Cardiology position paper, the reproductive health requirements of women with RHD demands urgent attention, shared informed decision making, multidisciplinary cooperation, and integration of relevant services to ensure the best possible perinatal and preconception care of women [80]. 

## 7. Management & Prevention

The management of ARF consists of eradication of the GAS using antibiotic (penicillin or alternative in penicillin allergic individuals) and symptomatic relief with anti-inflammatory drugs, analgesics, and bed rest. Secondary prophylaxis to prevent new episodes of ARF is recommended for patients with previous ARF or established RHD patients, as they are at higher risk of GAS infections [80]. In its advanced stages, RHD causes considerable morbidity and premature mortality mainly related to low awareness of health professionals, low health literacy of patients/parents/carers, long distances to health facilities, low provision diagnostics and drugs, and unavailability of interventions (cardiac catheterization and/or surgery). The different levels of prevention are summarized in the Figure 1. 

Major challenges for the management of ARF and RHD in under-resourced settings include case identification, preoperative assessment, choice of procedure, and postoperative care. Resource allocation toward raising awareness in affected communities and active adequate and continuous training of the health professionals at all levels in the recognition, prevention, and management of GAS infections, ARF, and RHD is essential for any control program. RHD management includes the medical treatment of heart failure and other complications, correction of individual valve lesions through catheter-based Interventions for valve repair or replacement (e.g., commissurotomy for MS), as well as open heart surgery for valve repair or replacement with the performance of minimally invasive surgery wherever available. The decision is determined by several factors viz: age, gender, number, and severity of affected valves, as well as socioeconomic and geographic factors that may influence follow-up, access to anticoagulation, and adherence to long-term prophylaxis. 

Anticoagulation is indicated in patients with prosthetic valves and/or atrial fibrillation. Prevention of infective endocarditis in endemic regions is mostly through prevention of skin and oral infections and antibiotic prophylaxis prior to invasive procedures that might cause bacteriemia.

A safe and effective vaccine against ARF would be highly advantageous. Although such developments started in the early 1960s, progression towards a protective vaccine has been hampered by the widespread diversity of GAS strains (numerous *emm* types), cross-reactivity between streptococcal and host proteins, and lack of relevant animal models for studying the pathogenesis of RHD [81]. The three major types of vaccines are currently in development are those based on cell surface proteins, secreted proteins, and carbohydrates [82]. 

## 8. Ways Forward

ARF and RHD are neglected diseases caused by GAS that affect the poorest in the world, causing profound disability and death despite being preventable. Efforts to reduce the burden of ARF/RHD should focus not only on strengthening national health systems in endemic areas, but also on the creation of multisectoral initiatives targeting all stakeholders (including but not exclusive to patients and their associations, health professionals, basic scientists, researchers, community leadership, civil society organizations, and local government and funders), as well as prioritising high-risk groups such as schoolchildren and women of childbearing age. Addressing the socioeconomic determinants of health and implementing population-based disease control programs would have a significant impact on achieving the goal of eradicating ARF and RHD. The recent momentum from the renewed global interest in GAS-related diseases should be capitalized on. Another priority is training and equipping health care professionals at all levels with the skills and tools to recognize and manage GAS infections, ARF, and RHD. Finally, national programs with notification of ARF/RHD as well as decentralization and integration of care are important, as they have the potential to highlight GAS as a cause of chronic disease in children and adolescents, thus supporting efforts to prioritise them among the various streptococcal conditions in endemic regions, as part of our march towards universal health coverage.

## Data Availability

Not applicable.
